# Emergency open surgery with a duodenotomy and successful removal of an impacted basket following a complicated endoscopic retrograde cholangiopancreatography procedure: a case report

**DOI:** 10.1186/s13256-020-02608-1

**Published:** 2021-02-23

**Authors:** Ibrahim Abu Shakra, Maxim Bez, Amitai Bickel, Mahran Badran, Fahed Merei, Samer Ganam, Walid Kassis, Eli Kakiashvili

**Affiliations:** 1 Department of Surgery A, Galilee Medical Center, 22100 Nahariya, Israel; 2 Medical Corps, Israel Defense Forces, Ramat Gan, Israel; 3 Faculty of Medicine in the Galilee, Bar-Ilan University, Safed, Israel

**Keywords:** Impacted basket, Complications, Endoscopic retrograde cholangiopancreatography

## Abstract

**Background:**

Current management of choledocholithiasis entails the use of endoscopic retrograde cholangiopancreatography (ERCP) and clearance of the common bile duct. A rare complication of this procedure is the impaction of the basket by a large stone, which necessitates lithotripsy. Here we report a case of an impacted basket during ERCP, which was managed by open surgery with a duodenotomy and the manual removal of the basket.

**Case presentation:**

A 79-year-old Caucasian man was admitted to our department with yellowish discoloration of urine, skin and eyes. Abdominal ultrasonography showed a slightly thickened gallbladder, multiple gallbladder stones, dilated intrahepatic bile ducts and extrahepatic bile extending to 1.1 cm. A computed tomography (CT) scan demonstrated a stone in the common bile duct, which caused dilation of the biliary ducts. The patient was diagnosed with obstructive jaundice secondary to choledocholithiasis; and underwent an ERCP, a sphincterotomy and stone extraction. Four days following discharge, the patient was readmitted with jaundice, abdominal pain, vomiting and fever. He was diagnosed with ascending cholangitis and treated initially with antibiotics. A second ERCP revealed a dilated common bile duct and choledocholithiasis. Stone removal with a basket failed, as did mechanical lithotripsy. Finally, the wires of the basket were ruptured and stacked in the common bile duct together with the stone. During exploratory laparotomy, adhesiolysis, a Kocher maneuver of the duodenum and a subtotal cholecystectomy were performed. Choledochotomy did not succeed in removing the impacted wires together with the stone. Therefore, a duodenotomy and an extension of the sphincterotomy were performed, followed by high-pressure lavage of the common bile duct to remove additional small biliary stones. The choledochotomy and duodenotomy were closed by a one-layer suture, and a prophylactic gastroenterostomy was performed to prevent leakage from the common bile duct and the duodenum. The postoperative course was satisfactory.

**Conclusions:**

This is the first report in the literature of removal of an impacted Dormia basket through the papilla by performing a duodenotomy and an extension of the sphincterotomy, followed by gastroenterostomy.

## Background

Endoscopic retrograde cholangiopancreatography (ERCP) is currently the most effective treatment for the management of choledocholithiasis [1]. Complications of ERCP include pancreatitis, bleeding following sphincterotomy and infections. After sphincterotomy, a basket is often used to extract large gallstones from the common bile duct. In many instances, however, the extracted stone is too large to be removed. In rare cases, the extraction basket can become impacted within the bile duct as a result of a large stone diameter and a distal duct size discrepancy. Several lithotripsy techniques have been developed to break the stone and release the basket. These include mechanical, extracorporeal shock wave, and electrohydraulic and laser lithotripsy [2]. In rare cases, a surgical intervention is required to remove the basket. We present a particularly difficult case of stone removal, which included a duodenotomy and a gastroenterostomy.

## Case presentation

We report a 79-year-old Caucasian man with a history of dementia, ischemic heart disease, diabetes mellitus, hypertension and cerebrovascular accident. He was admitted to our department with yellowish discoloration of urine, skin and eyes, which had developed over the past few days. On physical examination, the patient was jaundiced and afebrile, with normal vital signs. There were no abdominal findings on clinical examination, and there were no clinical features of sepsis upon presentation. His laboratory investigations revealed serum total bilirubin 10 mg/dL, direct bilirubin 7.4 mg/dL, alkaline phosphatase 405 u/L, gamma-glutamyltransferase 223 u/L, aspartate transaminase 75 u/L and alanine transaminase 95 u/L. His inflammatory markers were within normal limits.

Abdominal ultrasonography showed a slightly thickened gallbladder, multiple gallbladder stones, dilated intrahepatic bile ducts and extrahepatic bile extending to 1.1 cm. A computed tomography (CT) scan of the abdomen and pelvis following oral and intravenous contrast administration demonstrated a stone in the common bile duct (CBD), which caused dilation of the biliary ducts. A diagnosis of obstructive jaundice secondary to choledocholithiasis was made, and the patient proceeded to an ERCP, sphincterotomy and stone extraction. After improvements in the patient’s clinical and laboratory conditions, and considering his comorbidities, a decision was made to avoid surgical intervention (cholecystectomy) and to provide symptomatic treatment only at this stage.

**Fig. 1.**
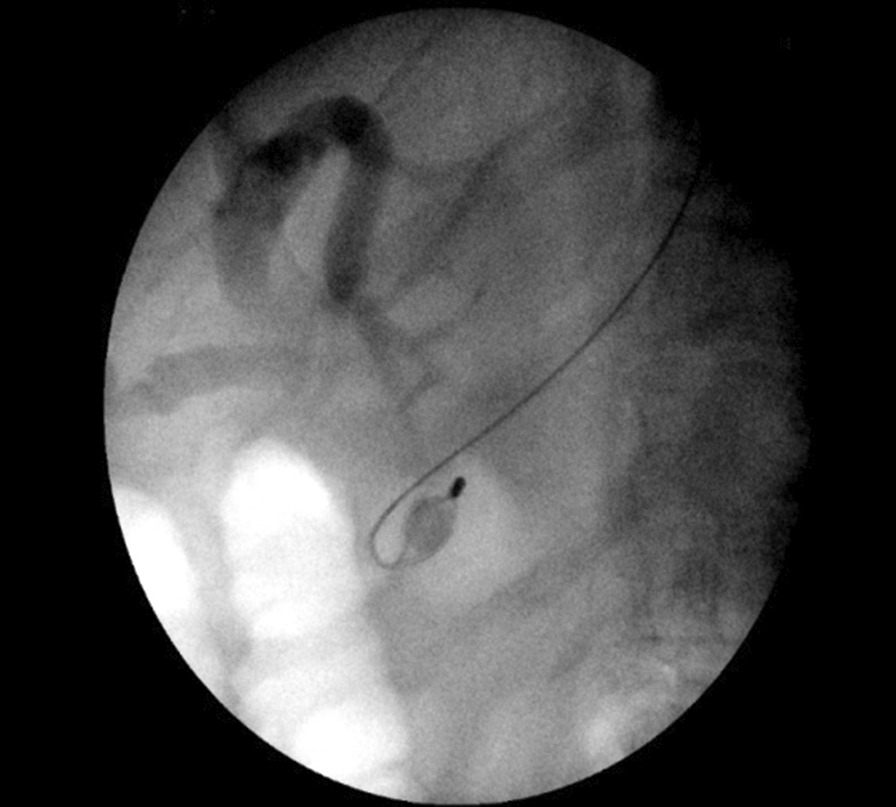
Endoscopic retrograde cholangiopancreatography showing an impacted Dormia basket in the common bile duct with a stone

**Fig. 2.**
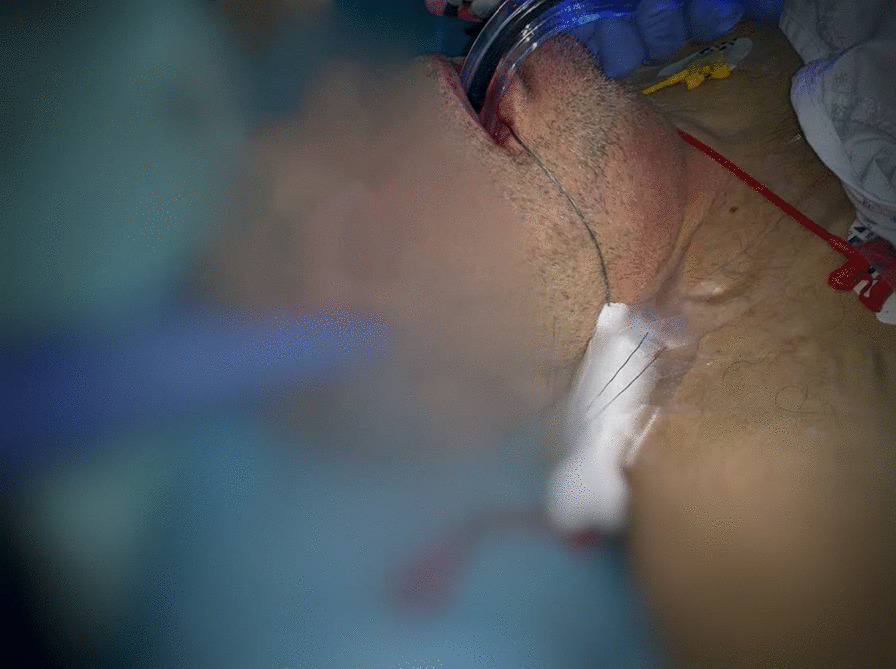
The cut edge of the basket handle can be seen emerging from the mouth, following failed removal of the basket

**Fig. 3.**
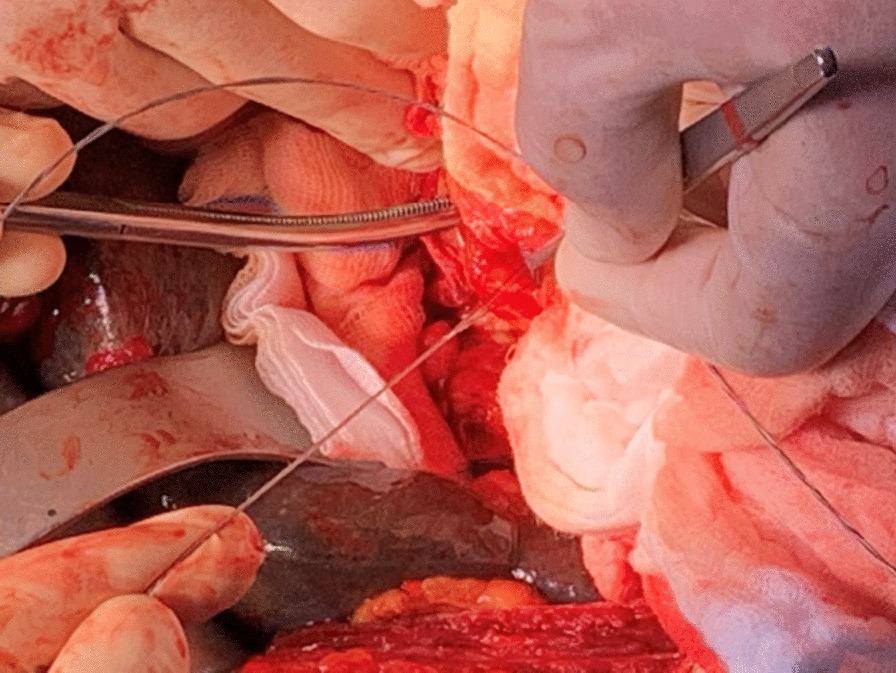
Extraction of the metal wires and the basket after performing duodenotomy

**Fig. 4.**
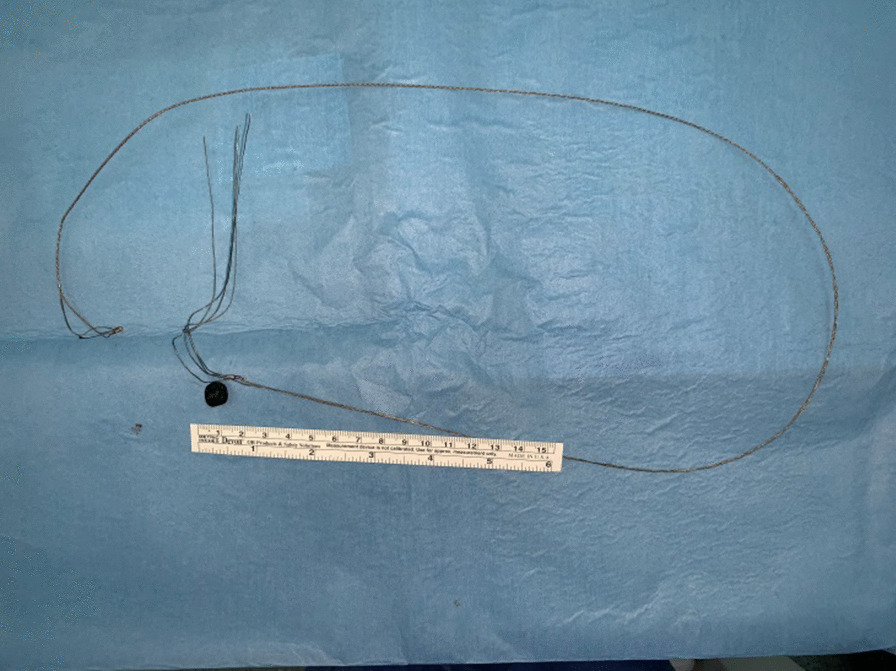
The retrieved basket with the stone

Four days following discharge, the patient was readmitted with jaundice, abdominal pain, vomiting and a fever of 38.3 °C. A diagnosis of ascending cholangitis was made and the patient was treated initially with antibiotics. A second ERCP was performed, which revealed a dilated CBD and choledocholithiasis (Fig. 1). An initial attempt of stone removal with a basket failed, and a mechanical lithotripsy to release the basket also failed. Finally, the wires of the basket were ruptured and stacked in the CBD together with the stone. Therefore, a decision for surgical treatment was made, and the patient was transferred to the operating room. During exploratory laparotomy (Fig. 2), adhesiolysis, a Kocher maneuver of the duodenum and a subtotal cholecystectomy were performed. Choledochotomy was done to directly remove the impacted wires together with the stone, but without success. Therefore, a duodenotomy and an extension of the sphincterotomy were performed to remove the impacted wires and the stone in the CBD (Figs. 3, 4). By means of a high-pressure lavage of the CBD, additional small biliary stones were removed. The choledochotomy and duodenotomy were closed by one-layer suture, and a prophylactic gastroenterostomy was performed as a protection, to prevent leakage from the CBD and the duodenum. The postoperative course was satisfactory; the patient recovered well from the surgery, and was discharged at 7 days postoperative. A 6-month follow-up was satisfactory without complications.

## Discussion and conclusions

ERCP is a diagnostic and therapeutic procedure used to treat biliary and pancreatic diseases. ERCP can successfully remove bile duct stones with a classical Dormia basket or balloon catheters in 85–95% of patients [3]. The common complications arising from ERCP are well recognized and include acute pancreatitis, bleeding, perforation and cholangitis. Post-ERCP pancreatitis is the most common serious complication [4], with an incidence of approximately 3.5% [5]. Hemorrhagic complications during or after ERCP occur in 1–2% of patients [6]. Perforations occur in 0.3% to 0.6% of ERCP cases [6]. Infectious complications of post-ERCP that present as cholangitis and cholecystitis occur in 0.5–3% of patients [6].

Basket impaction is a rare and unusual complication during ERCP that can occur after an attempt to remove biliary stones with a basket. This is a potentially dangerous complication during the ERCP procedure. It is characterized by an inability to withdraw the basket with the stone from the papilla or to separate the stone from the basket within the biliary duct. Several kinds of baskets made from metal wires are available in a variety of sizes and configurations for stone extractions. A Dormia basket may fail in the presence of a large stone (> 1 cm). This is the main factor responsible for impaction of the basket [2]. Impaction of a biliary basket due to a large stone was reported in 0.8–6% of cases [2]. However, due to advances in therapeutic techniques for CBD stones, this incidence has decreased to 0.8%. Risk factors reported for impaction of the biliary basket are inadequate dilation of the CBD and insufficient space between the stone and the CBD wall to open the wire basket [7].

Since only single documented reports of basket impaction have been reported, the optimal approach to treatment has not been established. At present, both endoscopic and surgical approaches are attempted to remove the basket. Endoscopic removal was reported using a second basket [8] or forceps [9]. However, these procedures require experienced endoscopists and sophisticated technological equipment. The percutaneous transhepatic method is also a minimally invasive method and can be used if the endoscopic method fails. However, this causes additional trauma to the liver and biliary radicals, which may lead to bile leakage, abscess formation and sepsis. Since the majority of the affected patients would benefit from cholecystectomy, a surgical approach should be considered for dealing with an impacted basket. As described in the present case report, our approach combined the removal of the impacted basket with a cholecystectomy. A choledochotomy should be first attempted to remove the basket. In this report, the surgical team was unable to remove the basket with the impacted stone and opted to remove it through the papilla by performing a duodenotomy and an extension of the sphincterotomy, followed by a gastroenterostomy. To the best of our knowledge, this is the first report to describe such an approach for the removal of an impacted basket.

## Data Availability

Not applicable.

## Abbreviations

ERCP: Endoscopic retrograde cholangiopancreatography
CBD: Common bile duct
